# A comparative review on neuroethical issues in neuroscientific and neuroethical journals

**DOI:** 10.3389/fnins.2023.1160611

**Published:** 2023-09-14

**Authors:** Shu Ishida, Yu Nishitsutsumi, Hideki Kashioka, Takahisa Taguchi, Ryuma Shineha

**Affiliations:** ^1^Graduate School of Life Sciences, Tohoku University, Sendai, Japan; ^2^Center for Information and Neural Networks, National Institute of Information and Communications Technology, Suita, Japan; ^3^Research Center on Ethical, Legal, and Social Issues, Osaka University, Suita, Japan

**Keywords:** responsible research and innovation (RRI), ethics integration, literature review, comparative analysis, neuroethical journals, neuroscientific journals

## Abstract

This study is a pilot literature review that compares the interest of neuroethicists and neuroscientists. It aims to determine whether there is a significant gap between the neuroethical issues addressed in philosophical neuroethics journals and neuroscience journals. We retrieved 614 articles from two specialist neuroethics journals (*Neuroethics* and *AJOB Neuroscience*) and 82 neuroethics-focused articles from three specialist neuroscience journals (*Neuron*, *Nature Neuroscience*, and *Nature Reviews Neuroscience*). We classified these articles in light of the neuroethical issue in question before we compared the neuroethical issues addressed in philosophical neuroethics with those addressed by neuroscientists. A notable result is a parallelism between them as a general tendency. Neuroscientific articles cover most neuroethical issues discussed by philosophical ethicists and vice versa. Subsequently, there are notable discrepancies between the two bodies of neuroethics literature. For instance, theoretical questions, such as the ethics of moral enhancement and the philosophical implications of neuroscientific findings on our conception of personhood, are more intensely discussed in philosophical-neuroethical articles. Conversely, neuroscientific articles tend to emphasize practical questions, such as how to successfully integrate ethical perspectives into scientific research projects and justifiable practices of animal-involving neuroscientific research. These observations will help us settle the common starting point of the attempt at “ethics integration” in emerging neuroscience, contributing to better governance design and neuroethical practice.

## 1. Introduction

This study is a comparative literature review in the field of neuroethics—i.e., “an interdisciplinary field focusing on ethical issues raised by our increased and constantly improving understanding of the brain and our ability to monitor and influence it” (Roskies, 2021). In this paper, we aim to compare the focus of two academic camps that comprise this interdisciplinary field: neuroscientists and neuroethicists. Our central question is what kind of differences (and similarities) can be found in the interest of the two parties when they discuss various neuroethical issues. For instance, is there any ethical question that has attracted the disproportionate attention of philosophical neuroethicists compared to neuroscientists, or vice versa? Such observations not only provide an understanding of the current state of the art of neuroethics but also help us settle the common starting point of the attempt at ethics integration, i.e., the “process by which scientists and ethicists engage with each other (…) to understand the social and ethical dimensions of (neuroscientific research), including the relationship between science and the societal context in which it operates” (United States Presidential Commission for the Study of Bioethical Issues, 2014, 12).

The notion of ethics integration is gradually more salient in neuroscience due to the increasingly complicated nature of neuroethical issues. In particular, the range of “neuroethical issues”—ethical issues involved in neuroscience and its application, broadly construed—has expanded and been intermingled with other disciplines such as information and communication technology (ICT), artificial intelligence (AI), medicine, genetics, tissue engineering, et cetera. This trend is significant, especially compared to the earliest days of “neuroethics” (Roskies, 2002). Along with this growing complication of neuroethics, there have been international efforts to tackle various neuroethical issues through active engagement between neuroscientists and neuroethicists (Zimmer, 2021), where the field of neuroethics is seen as “a mutually informing collaborator that can advance the field of neuroscience” (Amadio et al., 2018, 19). In addition, much attention has been paid to the governance design of neuroscience and its related neurotechnology, including the US Presidential Bioethics Commission’s two-volume report, *Gray Matters* (United States Presidential Commission for the Study of Bioethical Issues, 2014, 2015), and OECD’s brief document, *Recommendation of the Council on Responsible Innovation in Neurotechnology* (OECD, 2019), among others. Notably, the two major neuroscientific research projects—the BRAIN Initiative of the United States and the Human Brain Project of the European Union—have made efforts to “integrate” ethical perspectives into scientific research practice since its earliest stage (Ramos et al., 2019; Salles et al., 2019a), partly measuring up to the idea of responsible research and innovation (RRI) (Stilgoe and Guston, 2017; Stahl et al., 2021).

The attempt at “ethics integration” is fostered through constant and effective communication between two relevant communities: neuroscientists and neuroethicists. However, a potential obstacle to this attempt is the lack of interdisciplinary communication between the two academic communities. Specifically, there is not always a substantial consensus between neuroscientists and neuroethicists about what “neuroethical issues” are urgent, salient, and worth discussing. On reflection, it is unsurprising that not all neuroethical issues that are philosophically fascinating attract the concern of scientists or that not all neuroethical issues that scientists sincerely care about fit the existing philosophical literature.

In order to encourage and improve the interdisciplinary communication between neuroethics and neuroscience, it is necessary to examine the current situation and see to what degree the two communities share concerns on various neuroethical issues. Such examination will provide a common starting point for neuroethical discussion across the disciplines is required.

Here, we conducted a comparative analysis between neuroethics journals and neuroscience journals. We collated the neuroethical issues addressed in the two lines of “neuroethics discourse.” Section 2 describes the method of our comparative study and the resources exploited therein. Section 3 illustrates the results, which include general and specific findings on what neuroethical issues are discussed in the two bodies of neuroethics literature. Section 4 provides our discussion based on the results, followed by some notes on the limitations and possible future extensions of this study. Section 5 presents the conclusion.

## 2. Materials and methods

### 2.1. Article selection

**FIGURE 1:**
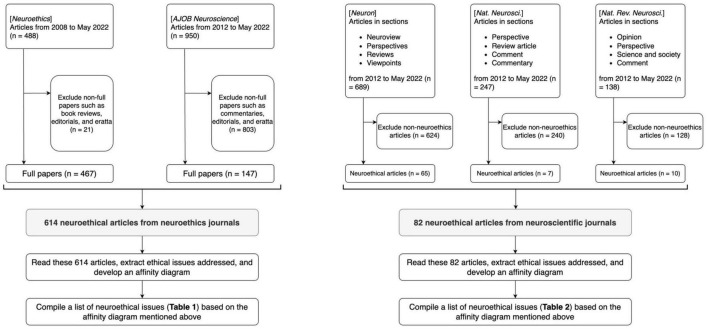
The process of article selection and data extraction. **Left:** the article selection process for neuroethics articles. **Right:** the article selection process for neuroscientific articles.

A literature search was conducted. We used the electronic database of two major philosophical neuroethics journals (*Neuroethics* and *AJOB Neuroscience*) and three major neuroscience journals (*Neuron*, *Nature Neuroscience*, and *Nature Reviews Neuroscience*).^1^ Figure 1 illustrates the process of article selection and data extraction.

As for the philosophical neuroethics journals (for brevity, call them *NE journals*), we collected all non-retracted research articles (call them *NE articles*), excluding editorials or other short articles such as “open peer commentaries” from *AJOB Neurosci*. We had 614 NE articles (467 from *Neuroethics*, 147 from *AJOB Neurosci.*) from the first to the latest issue as of May 2022 (*Neuroethics*: 2008–2022, *AJOB Neurosci.*: 2010–2022).

As for the neuroscientific journals (call them *NS journals*), conversely, we collected only ethics-focused articles (call them *NS articles*) from 2012 to May 2022. By NS articles, we mean articles that focus not on scientific findings (as regular scientific articles are) but on ethical issues concerning them, often under special sections such as “Neuroview” of *Neuron*, “Perspective” of *Nat. Neurosci.*, and “Science and Society” of *Nat. Rev. Neurosci*. Since not all articles in such sections are ethics-focused, screening was needed according to their title and abstract by two independent raters. Disagreement in this screening was resolved by discussion and consensus. In total, we had 82 NS articles (65 from *Neuron*, 7 from *Nat. Neurosci.*, and 10 from *Nat. Rev. Neurosci.*).

A note on article selection is in order. Neuroethical issues are also addressed in “general” journals—general philosophical-ethical journals like *Ethics* or general scientific journals like *Science*. However, this study focused only on five “specialist” journals, as mentioned above (see also Section “4.4. Limitations”). That is acceptable, given the purpose of the study. We do not mean to present a comprehensive list of neuroethical issues discussed in the field of ethics or science. The primary aim is to provide a view concerning discrepancies (or correspondence) between philosophical neuroethics and neuroscience concerning their coverage of various neuroethical issues.

### 2.2. Data extraction

In the next step, full-text articles were retrieved. Each paper was read and categorized according to the relevant neuroethical issues. The authors, who are experts in the field of neuroethics or neuroscience, developed two affinity diagrams—also known as the KJ method; see Scupin (1997)—to classify the abovementioned publications; one of the two diagrams is based on NE articles, and the other is based on NS articles. Correspondingly, disagreement on classification was resolved by discussion and consensus.

Following each affinity diagram, we compiled a list of neuroethical issues discussed in the relevant literature.^2^ Thus, we have two lists: Table 1 (derived from NE articles) and Table 2 (derived from NS articles) in the following section. In short, the primary task of this study is to compare the two lists and thereby contrast neuroethical issues covered in NE journals with those covered in NS journals.

**TABLE 1:** Neuroethical issues addressed in neuroethics journals.

Issue category	Issue	Description	Major keywords	References
Bias and diversity	Vulnerable social groups	Social groups that can be vulnerable to bias and/or discrimination as a result of neuroscientific findings	Sexism, neurodiversity	Gupta, 2012; Fine, 2013; Sarrett, 2016
Open science and innovation	Data governance	Issues about the ethically desirable treatment of neuroscientific data	Brain data, big data, mental privacy, sensitive data	Heinrichs, 2012; Kellmeyer, 2021
	Business and enterprise	Issues about business application of neuroscientific findings	Direct-to-consumer technology, neuromarketing, stakeholders	Chancellor and Chatterjee, 2011; Kreitmair, 2019; MacDuffie et al., 2022
Policy and governance	Policy-related issues	General issues about neuroscience-related policy	Regulation, human rights, neurorights, public mental health, political manipulation	Dubljević, 2015; Paulo and Bublitz, 2019; Goering et al., 2021; Ligthart et al., 2021
	Public engagement and public perception	Issues about public engagement related to neuroscience	Public attitudes, alternative medicine	Racine and Bell, 2012; Cabrera et al., 2015a; Nagappan et al., 2021
	Ethics integration	Issues about neuroscientific research initiatives and projects	Human Brain Project, BRAIN Initiative	Salles et al., 2019b; Goering and Klein, 2020
General bioethical issues	Research ethics	Bioethical issues concerning the research practice of neuroscience	Dual use, responsible research and innovation (RRI), informed consent	Dunn et al., 2011; Salles et al., 2019b; Goering et al., 2021
	Clinical ethics	Bioethical issues concerning clinical medical practice	Diagnosis, informed consent, end-of-life decision, surrogate decision, personhood, treatment, neurosurgery, side effects, personalized medicine, care	Jotterand et al., 2010; Hess, 2012; Bendtsen, 2013; Brukamp, 2013; Rommelfanger, 2013; Walker et al., 2022
	Animal ethics	Bioethical issues concerning animal sufferings	Animal experiments, veganism	Shriver, 2009; Johnson, 2020
	The ethics of enhancement	Bioethical issues concerning human neuro-enhancement	Moral enhancement, physical enhancement, sports, cognitive enhancement, psychiatric application, classroom application, mood enhancement, affective enhancement	Forlini and Racine, 2009; Singh and Kelleher, 2010; Kraemer, 2011; Lucke et al., 2011; Dubljević, 2013a; Schaefer et al., 2014; Focquaert and Schermer, 2015; Choy et al., 2020
	Other bioethical issues	General bioethical issues	Privacy, wellbeing, resource allocation, risk	Graham et al., 2015; Ligthart et al., 2021; Peterson et al., 2021; Fernandez et al., 2022
Implications for ethics	Theoretical issues	The application of neuroscientific findings for theoretical issues of ethics	Neuroscience of ethics, dual-process theory; free will, determinism, identity; agency; authenticity; autonomy	Kaposy, 2009; Appiah, 2010; Lavazza and De Caro, 2010; Felsen and Reiner, 2011; Baylis, 2013; Bluhm, 2014; Decety and Cowell, 2015; Gilbert, 2015a; Goddard, 2017; Goering et al., 2017; Saigle et al., 2018; Gilbert et al., 2021
	Practical issues	Issues about the application of neuroscientific findings focused on our free will and moral responsibility	Criminal justice, evidence, punishment, rehabilitation, psychopathy	Morse, 2008; Vincent, 2011; Klaming and Haselager, 2013; Pereboom, 2020; McCay and Kennett, 2021
Specific topics	Neurotechnologies	Issues related to a particular neurotechnology	Neuroimaging, tDCS, implant, BCI, extended mind, DBS, pills, head transplantation, brain organoid	Roskies, 2008; Klaming and Haselager, 2013; Dubljević, 2015; Zohny, 2015; Wolpe, 2017; Friedrich et al., 2021; Sawai et al., 2022
	Mental activities and psychiatric disorders	Issues targeted at particular neurological diseases or mental features	Addiction, dementia, locked-in syndrome, memory, belief, empathy, consciousness	Walter, 2010; Pickard, 2012, 2017; Fischer and Truog, 2013; Hammer et al., 2013; Bortolotti et al., 2014; Darby et al., 2016; Lewis, 2017; Goldberg, 2020

**TABLE 2:** Neuroethical issues addressed in neuroscience journals.

Issue category	Issue	Description	Major keywords	References
Bias and diversity	Vulnerable social groups	Social groups that can be vulnerable to bias and/or discrimination as a result of neuroscientific findings	Racism, neurodiversity, cultural diversity	Alivisatos et al., 2012; Hart, 2020
	Diversity in research community	Issues about discrimination and/or inequality among neuroscience researchers	Diversity, research community	Salinas and Bagni, 2017; Jones-London, 2020; Palser et al., 2022
	Diversity in non-research community	Issues about discrimination and/or inequality among the beneficiaries of neuroscience	Underrepresented patients, clinical trials	Boden-Albala, 2022; Webb et al., 2022
Open science and innovation	Data governance	Issues about the ethically desirable treatment of neuroscientific data	Data sharing, data access, data life-cycle management, sensitive data	Wiener et al., 2016; Salles et al., 2019a; Eke et al., 2022
	Business and enterprise	Issues about business application of neuroscientific findings	Enterprise, direct-to-consumer technology, responsible innovation	Garden et al., 2016; Coates McCall et al., 2019
Policy and governance	Policy-related issues	General issues about neuroscience-related policy	Regulation, human rights, neurorights, policymaking public interest	Schacter and Loftus, 2013; Dubljević et al., 2014; Glenn and Raine, 2014; Purcell and Rommelfanger, 2015; Farah, 2018; Hart, 2020
	Public engagement and public perception	Issues about public engagement related to neuroscience	Public attitudes, attitudes to animal research, education	Bennett and Ringach, 2016; Roskams and Popović, 2016; Gage, 2019
	Ethics integration	Issues about neuroscientific research initiatives and projects	International Brain Initiative, Human Brain Project, BRAIN Initiative	Rose, 2014; Amadio et al., 2018; Ramos et al., 2019; Adams et al., 2020
General bioethical issues	Research ethics	Bioethical issues concerning the research practice of neuroscience	Dual use, responsible research and innovation (RRI), informed consent	Ienca et al., 2018; Salles et al., 2019a; Sierra-Mercado et al., 2019; Feinsinger et al., 2022
	Clinical ethics	Bioethical issues concerning clinical medical practice	Informed consent	Feinsinger et al., 2022
	Animal ethics	Bioethical issues concerning animal sufferings	Animal experiments	Bennett and Ringach, 2016; Homberg et al., 2021
	The ethics of enhancement	Bioethical issues concerning human neuro-enhancement	Enhancement	Farah, 2015; Amadio et al., 2018
	Other bioethical issues	Other bioethical issues	Privacy, human rights, inequality, discrimination	Alivisatos et al., 2012; Ienca et al., 2018; Eke et al., 2022
Implications for ethics	Theoretical issues	The application of neuroscientific findings for theoretical issues of ethics	Ethics, moral decisions	Kelly and O’Connell, 2020
	Practical issues	Issues about the application of neuroscientific findings focused on our free will and moral responsibility	Criminal justice, evidence, punishment	Schacter and Loftus, 2013; Appelbaum, 2014; Galván, 2014
Specific topics	Neurotechnologies	Issues related to a particular neurotechnology	Wearable, neuroimaging, tDCS, intracranial neuroscience, implant	Dubljević et al., 2014; Coates McCall et al., 2019; Shen et al., 2020
	Mental activities and psychiatric disorders	Issues targeted at particular neurological diseases or mental features	Addiction, consciousness	Humphreys, 2019; Owen, 2019

## 3. Results

Tables 1, 2 show the main results of our study. In the following, we first demonstrate the general findings from comparing the two tables, i.e., the recent trend in what neuroethical issues have been presented and discussed in the relevant philosophical and scientific literature (Section “3.1. General findings”). Next, we advance to specific findings concerning particular issues (Section “3.2. Issue-specific findings”).

### 3.1. General findings

The literature research shows that a considerable number of articles in neuroscience journals address ethical issues that are broadly construed. The majority of such articles—65 out of 82—appear in the “Neuroview” section of *Neuron*.

Our results are shown in Tables 1, 2. They reveal a substantial correspondence between neuroethical issues discussed in NE journals and NS journals. Roughly speaking, the neuroethical issues addressed in NE articles are also covered in NS articles, and vice versa. More detailed comparisons are provided in Section “3.2. Issue-specific findings.”

However, there are significant differences as well. For instance, some of the neuroethical issues discussed in many NE articles—e.g., implications of neuroscientific findings for the idea of free will (see Section “3.2.5. Implications of neuroscience for ethics”)—do not appear explicitly in NS articles. Furthermore, topics such as the ethical aspects of neuroenhancement (see Section “3.2.4. General issues of bioethics”) are discussed thoroughly in NE articles compared to NS articles. Others, such as international attempts at “ethics integration” (see Section “3.2.3. Policy and governance”) and the moral permissibility of animal experiments (see Section “3.2.4. General issues of bioethics”), are addressed in much more detail in NS articles than in NE articles.

### 3.2. Issue-specific findings

Based on the affinity diagrams we developed, we have six categories of neuroethical issues listed in the two tables. The categories include (1) bias and diversity; (2) open science and innovation; (3) policy and governance; (4) general issues of bioethics; (5) implications for ethics; and (6) other, more specific topics of neuroethics. In the following subsections, we describe issue-specific findings on what neuroethical issues are addressed in philosophical and scientific literature.

#### 3.2.1. Bias and diversity

The first category refers to neuroethical issues concerning bias, discrimination, and diversity. From the literature, three relevant lines of diversity-related issues can be found: *vulnerable social groups*, *diversity in the research community*, and *diversity in the non-research community*.

##### 3.2.1.1. Vulnerable social groups

Many NE articles address the concern that some social groups are vulnerable to discrimination due to the misapplication of neuroscientific findings. Such groups include LGBTQ people (Gupta, 2012)^3^ and disabled people (Sarrett, 2016). Nonetheless, in general, women and gender-related issues attract more attention. For instance, Roy (2012) emphasizes the point of productive engagement of feminist scholars with neuroimaging studies to further our ethical response to gender differences. Bluhm (2013) and Fine (2013) discuss the issue of “neurosexism,” i.e., the misuse of neuroscientific observations to justify and reinforce our problematic views of gender roles. In addition, Ienca and Ignatiadis (2020) point to the risk of “neurodiscrimination,” a new kind of discrimination against individuals based on their difference in neurocognitive features.

Such concerns appear in NS articles as well. Alivisatos et al. (2012) mention the potential ethical concern of neurodiscrimination related to a particular research project.^4^ Hart (2020) worries about discrimination against Black people through the over-exaggeration of the neuroscientific findings about drugs.

##### 3.2.1.2. Diversity in the research community

Neuroscientific articles pay much more attention to the diversity in the neuroscientific research community. Specifically, they focus predominantly on the over- and under-representation of various social groups in the community. In this trend, the under-representation of women (Salinas and Bagni, 2017; Dworkin et al., 2020; Llorens et al., 2021) and that of Black people (Jones-London, 2020; De Los Reyes and Uddin, 2021; Wheaton, 2021) are the two major topics. Palser et al. (2022) address the demographic imbalance in the editorial board of neuroscientific journals, focusing on gender and geographic disparity. Such concerns are sometimes linked to the view that the diversity in the research community has a good effect on the advance of scientific research (Asplund and Welle, 2018; Richardson et al., 2021). In NE articles, however, a comparable discussion is not found.

##### 3.2.1.3. Diversity in the non-research community

Neuroscientific articles address the under-representation of marginalized social groups in a different context. For instance, it is pointed out that Black people make up a disproportionately small share of the participants in research trials and/or experiments (Weinberger et al., 2020; Boden-Albala, 2022; Webb et al., 2022). To reiterate, this is a unique issue addressed in NS articles, contrasted with NE articles.

#### 3.2.2. Open science and innovation

Topics in the second category concern open science and innovation. Two kinds of ethical issues in this context are relevant here: *data governance* and *business and enterprise*.

##### 3.2.2.1. Data governance

Several NE articles address the ethical treatment of data related to brain activities (sometimes called “brain data” or “neurodata”). They highlight the allegedly exceptional feature of brain data, which is said to call for special and stricter protection (Heinrichs, 2012; Ienca and Ignatiadis, 2020; Kellmeyer, 2021).^5^ Partly motivated by this observation, the ethical concern about privacy is one of the most discussed topics of neuroethics in NE articles (see Section “3.2.4. General issues of bioethics”; cf. Salles et al., 2018; Garden et al., 2019; OECD, 2019).

A similar concern appears in NS articles, with their central focus on viable workflows and practices to protect such sensitive brain data. For instance, Salles et al. (2019a) illustrate the attempt by the Human Brain Project, and Eke et al. (2022) suggest an international framework of robust data governance. Notably, they refer to the relatively novel idea of “data life-cycle management.”

In addition to data protection, data *sharing* is another major issue addressed in NS articles. They highlight the importance of productive data-sharing for the progress of neuroscientific research (Teeters et al., 2015; Wiener et al., 2016; Eke et al., 2022). In order to establish a worldwide data-sharing framework compatible with privacy concerns (such as the anonymization of brain data), some articles illustrate actual practices, such as the Neurodata Without Borders initiative (Teeters et al., 2015), the Human Brain Project (Salles et al., 2019a), and the International Brain Initiative (Eke et al., 2022), while others involve policy recommendations (Wiener et al., 2016; Eke et al., 2022). Compared to such prominent attention to data sharing in NS articles, the corresponding discussion is significantly limited in NE articles.

##### 3.2.2.2. Business and enterprise

Many NE articles address ethical issues concerning the emerging “neurotech” industry and related business affairs. First, Kreitmair (2019) lays out major ethical considerations involved in the direct-to-consumer (DTC) neurotech markets, such as safety, transparency, privacy, and just distribution. Relatedly, Kellmeyer (2021) discusses the concern of safety and privacy accompanied by the big data collected through DTC neurotechnology. A different business-related issue concerns the ethics of “neuromarketing,” the application of neuroscientific findings to marketing strategy (Chancellor and Chatterjee, 2011; Daley and Howell, 2018; Shahriari et al., 2020). Bradfield (2021), referring to the problem of incidental findings, argues that neuromarketing researchers have similar ethical obligations as academic neuroscientists. Third, some papers investigate what various stakeholders think of ethical issues related to neurotechnology. Nijboer et al. (2013) display the opinions of BCI researchers, whereas MacDuffie et al. (2022) involve those of neurotech industry personnel and laypeople.

The findings reveal that similar questions are discussed in NS articles as well. First, ethical issues involved in DTC neurotechnology are assessed (Garden et al., 2016; Coates McCall et al., 2019). Second, some papers mention the idea of RRI, or sometimes simply “responsible innovation,” to discuss the ethical issues involved in neurotech business practices (Garden et al., 2016; Coates McCall et al., 2019). See also Section “3.2.4. General issues of bioethics” for more on RRI.

#### 3.2.3. Policy and governance

The third category pertains to a family of issues related to policy, regulation, and governance of neuroscientific practices. They are divided into the following three threads. One of the three is *policy-related issues*, which contain ethical issues regarding policy in a narrow sense. The other two concern policy-related issues in broader contexts: *public engagement and perception* and *ethics integration*, respectively.

##### 3.2.3.1. Policy-related issues

In various NE articles, potential regulatory frameworks are suggested or explored relative to particular neuroscientific topics, such as neuroenhancement methods (Pustovrh and Mali, 2014; Dubljević, 2015; Jwa, 2019), the usage of brain data (Kellmeyer, 2021), and brain organoids (Sawai et al., 2022). In a somewhat different context, Greenhow and East (2015) address ethical issues involved in the regulation of contact sports, citing neurological observations about the effect of concussions on athletes’ brains.

Another family of policy-related issues bears on human rights specifically relevant to neuroscience and its applications. Ligthart et al. (2021) discuss the idea of the right to mental privacy in the context of criminal justice. Craig (2016) elaborates on the notion of the right to mental integrity—as opposed to bodily integrity—that can be infringed by the misuse of brain intervention technologies. These kinds of human rights, among others, are sometimes called “neurorights,” indicating that such human rights are unique, in some sense, to neurotechnology (Goering et al., 2021). However, the theoretical and practical status of “neurorights” as a brand-new normative concept is under dispute (Bublitz, 2022).

Finally, several NE articles explore the possibility of applying neuroscientific findings to other policymaking issues. One such topic is public mental health (Mackenzie, 2011; Lucke and Partridge, 2013; Cratsley, 2019); another is the avoidance of political manipulation (Dubljević, 2013b; Paulo and Bublitz, 2019); and yet another is in the context of criminal justice and punishment (see Section “3.2.5. Implications of neuroscience for ethics”). A distinct but related topic is “neuroeducation”—the attempt to apply neuroscientific findings to educational practice (Ansari et al., 2012; Hardiman et al., 2012; Hook and Farah, 2013).

A remarkable parallel is found in NS articles. First, potential regulatory challenges are discussed relative to particular neuroscientific topics. They include, among others, neuroenhancement methods (Garden et al., 2016; Hart, 2020), the usage of brain data (Purcell and Rommelfanger, 2015; Eke et al., 2022), neurological medical devices (Dubljević et al., 2014; Anderson et al., 2016), and neurotechnologies with potential military purposes (Ienca et al., 2018).

Second, some articles focus on human rights related to neuroscience and its applications. Such rights include the right to mental privacy, mental integrity, and psychological continuity (Ienca et al., 2018; Eke et al., 2022). Farah (2015) is an exceptional case—she addresses practical questions about human rights involved in neurotechnology and offers theoretical foundations for the philosophical-ethical notion of rights.

Finally, like NE articles, some NS articles address the possibility of applying neuroscientific findings to other policy issues. One such topic is public mental health (Humphreys, 2019); another relates to criminal justice and punishment (Schacter and Loftus, 2013; Appelbaum, 2014; Galván, 2014; Glenn and Raine, 2014); and still another concerns the possibility that neuroscientific findings can contribute to socioeconomic equality (Farah, 2018).^6^ See also Section “3.2.5. Implications of neuroscience for ethics.”

##### 3.2.3.2. Public engagement and perception

Our findings establish that a considerable number of NE articles concern issues of public engagement in neuroscience—whether in neuroscientific research as such or neurotechnology-focused policymaking or regulation. Many of them are empirical studies on public attitudes and expectations toward neuroenhancement (Fitz et al., 2014; Cabrera et al., 2015a,b; Bard et al., 2018; Dinh et al., 2020). Whitehead and Chandler (2020) explore public attitudes to neurorehabilitation—neurological intervention to reduce the risk of recidivism. Racine and Bell (2012) analyze the overly optimistic press coverage of deep brain stimulation (DBS) and discuss its consequences. Finally, unlike these empirical studies, Nagappan et al. (2021) assess the emerging trend of “alternative neurotherapies,” or non-mainstream neurological therapies, from ethical and legal perspectives.

The importance of public engagement in neuroscience is also mentioned in NS articles, despite the abovementioned style of empirical surveys not being the primary way such articles take. For instance, Bennett and Ringach (2016) and Mendez et al. (2022) urge more public outreach of neuroscientific research involving animals, given that public support and understanding of animal experimentation is one of the most controversial issues that the leading neuroscientific research would have to tackle (see also Section “3.2.4. General issues of bioethics”). Another idea of public engagement is for laypeople to participate in scientific research in a substantial manner other than as mere subjects of experiments (Purcell and Rommelfanger, 2015; Roskams and Popović, 2016; Gau et al., 2021). Finally, Gage (2019) argues for education about neuroscience as early as in middle or high schools to widely disseminate the basic tools and knowledge of (the research into) our brains and their function.

##### 3.2.3.3. Ethics integration

The notion of ethics integration denotes “a process by which scientists and ethicists engage with each other (…) to understand the social and ethical dimensions of their work” (United States Presidential Commission for the Study of Bioethical Issues, 2014, 12).

Both NE and NS articles mention this notion to analyze actual research practices. However, the focus of NE literature is almost dominated by the “big two”—the Human Brain Project funded by the EU (Salles et al., 2019b; Salles and Farisco, 2020) and the BRAIN Initiative funded by the US government (Goering and Klein, 2020). Christen et al. (2016), while focusing on the Human Brain Project as a typical case, point to more general issues involved in giant research projects. Additionally, an exceptional article is by Moses and Illes (2017), who compare ethical committees in various professional organizations for neuroscientists.

In contrast, NS literature seems to have a much broader coverage of case studies. It includes not only the largest practices in Europe (Rose, 2014; Greely et al., 2016; Salles et al., 2019a) and the US (Greely et al., 2016; Ramos et al., 2019; Feinsinger et al., 2022) but also ones in Australia (Carter et al., 2019), Canada (Illes et al., 2019), China (Wang et al., 2019), Japan (Sadato et al., 2019), and South Korea (Jeong et al., 2019).^7^ These research practices are compared by Amadio et al. (2018) in terms of the ethical questions in which they engage. Finally, in comparison to these nationwide research projects, an international approach to ethics integration—the International Brain Initiative—is sketched (Amadio et al., 2018; Adams et al., 2020).

#### 3.2.4. General issues of bioethics

The fourth category covers a wide range of bioethical issues that are not necessarily specific to neuroscience and its applications. From our findings, the following five sub-categories are found: *research ethics*, *clinical ethics*, *animal ethics*, *the ethics of enhancement*, and others.

##### 3.2.4.1. Research ethics

Three major topics are present in both NE and NS literature. First, a familiar problem of dual use of neurotechnology—its unintended application for military purposes being a typical problem—appears in both NE articles (Goering et al., 2021) and NS articles (Rose, 2014; Ienca et al., 2018). Second, another common issue of protecting the rights of research participants, including but not limited to their voluntary participation with informed consent, is mentioned in both two kinds of paper. On the NE side, for instance, Dunn et al. (2011) discuss various consent-related ethical issues involved in deep brain stimulation (DBS) research applied to psychiatry, mainly focusing on the validity of consent by persons with mental illness. Mergenthaler et al. (2021) illustrate various perspectives of neuroscience researchers on how to recruit participants and obtain their consent. On the NS side, Feinsinger et al. (2022) suggest various ways to ensure the voluntariness of participation in neuroscientific research. Sierra-Mercado et al. (2019) discuss whether brain implant researchers have a moral obligation to cover the cost of device removal requested by participants when the removal is not necessary from a medical perspective. Finally, NE articles (Salles et al., 2019b; Goering et al., 2021), as well as NS articles (Rose, 2014; Garden et al., 2016; Salles et al., 2019), pay attention to the relatively new notion of RRI. With some disagreement in what specific issues are discussed and to what extent, there is no stark contrast in the ethical issues addressed in NE and NS articles.

##### 3.2.4.2. Clinical ethics

Informed consent is one of the typical ethical issues concerning clinical medicine as well. It is addressed in NE articles such as Jotterand et al. (2010) and Gilbert (2015b) as well as in NS articles such as Feinsinger et al. (2022).^8^ Beyond this point, however, the two bodies of literature have different focuses on clinical-neuroethical issues. Consider, first, ethical issues regarding medical trials. The central focus of NS articles in this context is the under-representation of vulnerable social groups in medical trials (Boden-Albala, 2022); see also Section “3.2.1. Bias and diversity.” NE articles, in contrast, address a broader range of issues, such as the moral justifiability of certain types of medical trials (Hess, 2012; Gilbert et al., 2014; Hurst et al., 2015) and the ethical usage of placebos (Rommelfanger, 2013). Other clinical-ethical issues addressed in NE articles include: personalized neurological medicine (Walker et al., 2022), surrogate end-of-life decision-making through using neurotechnology (Bendtsen, 2013; Fernández-Espejo and Owen, 2013; Schembs et al., 2021), ethical questions involved in diagnosis (Brukamp, 2013; Rodrigue et al., 2013; Cratsley, 2019), and the ethically desirable treatment for patients with disorders of consciousness (Brukamp, 2013; Farisco, 2013; Farisco et al., 2014; Lavazza and Reichlin, 2018; Peterson et al., 2021).

##### 3.2.4.3. Animal ethics

One focal issue is the ethics of animal experimentation. Given that the use of laboratory animals—including non-human primates—is often necessary to advance neuroscientific research, both NE (Eberwine and Kahn, 2020; Johnson, 2020) and NS (Roelfsema and Treue, 2014; Homberg et al., 2021) have confronted this problem. In this context, some NS articles highlight the importance of public engagement to foster public support for animal research (Bennett and Ringach, 2016; Mendez et al., 2022); see also Section “3.2.3. Policy and governance.”

Other animal-ethical issues are also contained in NE articles. For instance, Shriver (2009) examines the morality of genetically reducing the capacity of livestock to suffer, which could lead to practices of farming with less suffering. Basl (2010) discusses the problem involved in cognitive enhancement for non-human animals, which might alter the moral status of them relative to that of human beings. Finally, Timm (2016) applies the findings of cognitive science to analyze the disagreement in the animal ethics debate; see also Section “3.2.5. Implications of neuroscience for ethics.”

##### 3.2.4.4. The ethics of neuroenhancement

The ethical discussion about neurotechnology-based enhancement, or “neuroenhancement,” abounds among NE articles.

First, note that there is more than one type of neuroenhancement based on what function is enhanced by neurotechnology. One oft-heard type is cognitive enhancement. Some articles discuss “first-order” normative issues raised by cognitive enhancement, such as coerced enhancement (Dubljević, 2013a), the possible threat to autonomy or authenticity (Schaefer et al., 2014), distributive justice (Fröding and Juth, 2015), fair competition (Petersen and Lippert-Rasmussen, 2021), and cheapened achievement (Gordon and Dunn, 2021). Singh and Kelleher (2010) focus on ethical issues relevant to using cognitive neuroenhancement in young people. Others survey public attitude about it (Forlini and Racine, 2009; Fitz et al., 2014; Conrad et al., 2019; Dinh et al., 2020), while others critically assess how neuroethicists discuss cognitive enhancement (Lucke et al., 2011; Outram, 2012; Zohny, 2015).

Another popular type is moral enhancement. Prominently, some articles address ethical questions raised by moral enhancement, such as coerced or forced enhancement (Focquaert and Schermer, 2015; Choy et al., 2020), the possible threat to autonomy or authenticity (Clewis, 2017; Lara and Deckers, 2020), and the purported tension with having a virtuous character (Banja, 2011; Jotterand, 2011). Others examine public attitudes about it (Specker et al., 2017), while others are critical assessments and suggestions about the neuroethical discussion about moral enhancement (Shook, 2012; Raus et al., 2014).

Aside from these two major types, several articles focus on mood or affective enhancement, discussing ethical issues involved in it (Ravelingien et al., 2009; Kraemer, 2011) or surveying public attitudes toward it (Cabrera et al., 2015a).

These papers might be classified along a different dimension—the situations or sites in which neuroenhancement technology is applied. One group addresses the ethical issues involved in the educational application of neuroenhancement (Forlini and Racine, 2009; Enck, 2013; Vrecko, 2013), largely focused on using cognitive enhancers by university students. Another group deals with the ethics of psychiatric neuroenhancement (Synofzik et al., 2012; Ilieva, 2015), which is sometimes further applied to forensic psychiatry (Barn, 2019; Choy et al., 2020; Specker et al., 2020). Several papers focus on neuroenhancement by athletes, sometimes raising the problem of “neuro doping” (Mihailov and Savulescu, 2018; Petersen and Lippert-Rasmussen, 2021; Pugh and Pugh, 2021). Finally, the ethical questions involved in the military application of neuroenhancement are discussed through an empirical survey by Sattler et al. (2022).

Compared to such a broad range of ethical issues covered in these philosophical-neuroethical papers, NS articles seem to have paid much less attention to neuroenhancement. That said, Farah (2015) and Amadio et al. (2018) provide lists of general ethical issues raised by neuroenhancement, including issues of coercion, fairness, distributive justice, the possible threat to autonomy or authenticity, and the purported tension with genuine achievement. More particular issues raised by neuroenhancement include the media coverage of cognitive enhancement products (Dubljević et al., 2014), the data treatment involved in “brain training” programs (Purcell and Rommelfanger, 2015), the dual use issue concerning the military application of cognitive enhancement (Ienca et al., 2018), and the non-discriminatory regulation of mood enhancement drugs (Hart, 2020).

##### 3.2.4.5. Other bioethical issues

Here is a brief list of other miscellaneous bioethical issues discussed in NE and NS articles. They include concerns about privacy; wellbeing; distributive justice and resource allocation; risk–benefit comparison; equality and discrimination; and human rights.

#### 3.2.5. Implications of neuroscience for ethics

Our brain activities are seen as tightly connected to our mental status and agency. This assumption lays the foundation of the fifth category of neuroethical issues. They concern the potential implications of neuroscientific findings for our understanding of ethically relevant notions such as intention, deliberation, judgment, action, agency, responsibility, et cetera.

##### 3.2.5.1. Theoretical issues

Neuroethics articles discuss the possibility that neuroscientific findings contribute to the theoretical inquiry into ethical questions (Appiah, 2010; Machery, 2010). This sort of approach is sometimes called the “neuroscience of ethics” (Roskies, 2021, §3), a neighboring scholarly field of “ethics of neuroscience.” A typical topic in this context is Joshua Greene’s well-known dual process theory (Dean, 2010; Klein, 2011; Bluhm, 2014; Mihailov and Savulescu, 2018). Decety and Cowell (2015) discuss the relationship between neuroscientific observations concerning empathy and our notions of fairness and justice. Crisp and Kringelbach (2018) examine whether the philosophical idea of “higher pleasure” is compatible with recent neuroscientific evidence. Not so many NS articles address such issues, but Kelly and O’Connell (2020) suggest how neuroscience can influence our understanding of morality by unraveling the neural mechanism behind our morality.

In addition to the “neuroscience of ethics” approach, many NE articles concentrate on the theoretical-philosophical questions regarding the nature of agency and moral responsibility. A typical philosophical dispute in this context revolves around free will, with a particular focus on whether neuroscientific findings about our mental activities support determinism (Kaposy, 2009; Sie and Wouters, 2010; Shepard and Reuter, 2012; Saigle et al., 2018). Similarly, neuroscientific evidence is often compared with philosophical notions such as autonomy (Felsen and Reiner, 2011; Gilbert, 2015a; Niker et al., 2018; Pugh et al., 2021; Zuk and Lázaro-Muñoz, 2021), agency (Baylis, 2013; Goddard, 2017; Goering et al., 2017), and the felt alienation to “become another person” (Kraemer, 2013; Witt et al., 2013; Gilbert et al., 2017). In this trend, however, Lavazza and De Caro (2010) are critical of philosophers that hastily draw unfounded theoretical implications on human agency from empirical neuroscientific findings. A similar warning is raised by Gilbert et al. (2021). This type of theoretical-philosophical issue is, at least according to our review, not addressed in NS articles.

##### 3.2.5.2. Practical issues

In contrast to these theoretical issues, the practical relevance of neuroscience to our practice of criminal justice and punishment is more widely addressed. For instance, both NE articles (Vincent, 2011; Berlin, 2014; Morse, 2014) and NS articles (Schacter and Loftus, 2013; Appelbaum, 2014) assess the morality of using brain data as evidence in court. Second, the implications of neuroscientific findings for the ethical way of punishment are examined in NE articles (McCay and Kennett, 2021) and NS articles (Galván, 2014). Relatedly, an NE article by Klaming and Haselager (2013) discusses questions about the ethical and legal responsibility of those with brain implants. In addition to these issues, some NE articles examine the ethical theory of retributivism, paying little attention to neuroscience-specific issues (Caruso, 2020; Jeppsson, 2021), whereas others focus on the ethical treatment of psychopaths (Morse, 2008; Gillett and Huang, 2013; Hübner and White, 2016). Finally, the moral justifiability of mandatory rehabilitation for criminals with neurotechnology—sometimes called “neurorehabilitation”—is assessed as a philosophical-ethical question (Pereboom, 2020; Holmen, 2021).

#### 3.2.6. Specific issues of neuroethics

Each of the five categories is grouped by the relevant ethical issues. In contrast, here are two brief lists composed of various neuroscience-related topics and situations in which the abovementioned ethical issues arise (See the two tables for detail).

First, some articles focus on ethical issues related to specific neurotechnologies. They include neuroimaging; transcranial direct-current stimulation (tDCS); brain implants; brain–computer interfaces (BCI); deep brain stimulation (DBS); chemical neuro-interventions such as antidepressants like Prozac, moral enhancers like omega-3 supplementation, and cognitive enhancers like Ritalin; head transplantation; brain organoids; and direct-to-consumer neurotechnologies.

Second, neuroethics articles sometimes concern specific mental activities and psychiatric disorders that neuroscientific research focuses on. Addiction is one of the prominent topics in this category. Several NE articles critically examine the dominant view of addiction as a “brain disease” (Pickard, 2012; Hammer et al., 2013; Lewis, 2017). Pickard (2017) proposes a normative framework to support those with addiction. Other than addiction, NE articles address topics such as dementia or Alzheimer’s disease; locked-in syndrome; disorders of consciousness such as the minimally conscious state (MCS) and the persistent vegetative state (PVS); delusion; chronic pain; memory; emotion; and empathy.

Note, in particular, that these two lists cut across the previous five categories of neuroethical issues, as we have seen, at least in principle.

## 4. Discussion

The present comparative review provides an overview of what neuroethical issues are addressed in the academic discourse. In particular, the study contributes to the literature by focusing on the discrepancy (and correspondence, for that matter) in the neuroethical topics addressed between the two relevant bodies of the academic discourse—philosophical neuroethics (NE) on the one hand and neuroscience (NS) on the other.

### 4.1. General discussion

As shown in Section “3.1. General findings,” one of the most noteworthy findings of this study is that there is substantial correspondence between neuroethical issues discussed in the NE and NS literature. In particular, while it is understandable that NE journals pay attention to a wide range of neuroethical issues, many of them are also covered in NS journals. Perhaps contrary to our expectations, our analysis indicates that the neuroethical discourse among scientists—at least as apparent in major NS journals—may be no less mature than the corresponding neuroethical discourse in NE journals in terms of the variety of neuroethical issues covered. Also, the results may somewhat dilute the criticism often leveled against philosophical ethicists that they are focusing too much on futuristic and unrealistic scenarios that bear no relation to what science is doing.^9^

By highlighting such a correspondence, however, we do not intend to downplay or disregard the fact that some discrepancy exists between the two bodies of literature, as illustrated in Section “3.2. Issue-specific findings.” They often highlight different neuroethical questions in different contexts. For instance, NS articles cover general ethical issues related to neuroenhancement, whereas NE articles cover a much broader range of enhancement issues (see Section “3.2.4. General issues of bioethics” for detailed findings on this point); see also Section “4.2. Issue-specific discussion.” However, such differences seem relatively small compared to the general parallelism we found between NE and NS articles regarding the neuroethical topics addressed. Unexpectedly, the majority of issues discussed in philosophical neuroethics appear in NS journals, and vice versa.

### 4.2. Issue-specific discussion

What was the noticeable discrepancy between NE and NS discourses on neuroethics? First, as mentioned briefly, the ethics of (neuro-)enhancement is much more comprehensively discussed in the NE literature than in its NS counterpart.^10^ Among the limited number of NS articles dealing with the ethical aspects of neuroenhancement, almost all of them concern *cognitive* enhancement (Dubljević et al., 2014; Farah, 2015; Purcell and Rommelfanger, 2015; Amadio et al., 2018; Ienca et al., 2018), with an exception focusing on mood enhancement or recreational drugs (Hart, 2020). Lacking here is the ethical examination of the potential application of neuroscientific findings for *moral* enhancement, which is one of the most intensely discussed topics in philosophical-neuroethical studies; see Section “3.2.4. General issues of bioethics.”^11^

Second, the NE literature offers a broad and detailed neuroethical investigation related to “PIAAAS”—shorthand for personality, identity, autonomy, authenticity, agency, and self; see Section “3.2.5. Implications of neuroscience for ethics.” It seems to be a unique feature of the NE side of neuroethics in comparison to the NS side, which (imaginably) pays much less attention to such a theoretical or even metaphysical debate. However, some recent NE articles (Gilbert et al., 2021; Pugh et al., 2021) urge philosophers to engage in an evidence-based discussion on this topic. Their suggestion might broaden the scope of the “neuroscience of ethics,” an interdisciplinary attempt to investigate ethical ideas with robust neuroscientific evidence.

Third, clinical-ethical issues are more broadly addressed in the NE literature than in the NS literature (see Section “3.2.4. General issues of bioethics.”). This difference can be simply because *Neuron*, *Nat. Neurosci.*, and *Nat. Rev. Neurosci.* are neuroscience journals. Their main specialty is not in, say, neurology as a medical discipline. See also Section “4.4. Limitations.”

In short, the three groups of neuroethical issues listed above appear in the NE literature while missing (or much less significant) in the NS counterpart.

Simultaneously, however, there are some neuroethical issues of which the reverse is true. First, the NS journals we employed in this study have rich literature concerning case studies of neuroscientific research projects with various funding agencies worldwide (see Section “3.2.3. Policy and governance”). Relevant articles in this line of literature often offer recommendations or “tips” for better practices to integrate ethics into neuroscientific research. In contrast, a limited number of NE articles discuss the practices of the world’s two major projects—the European Human Brain Project (Salles et al., 2019b) and the BRAIN Initiative by the United States (Goering and Klein, 2020).

Second, diversity is one of the “hot” issues on the NS side of the neuroethical discourse. Their main concern is the under-representation of some social groups among researchers and research participants, and many NS articles report actual attempts and potential recommendations on this matter; see Section “3.2.1. Bias and diversity.” Admittedly, some might doubt it should count as a “neuroethical issue” in a strict sense rather than a general issue of research ethics instantiated in neuroscientific research. With this classificatory question notwithstanding, it is undeniable that the diversity of researchers and research participants is an indispensable ethical concern involved in neuroscientific research.

Third, and finally, the ethical justifiability of animal experimentation seems much more salient in the NS literature than in its NE equivalent. As seen in Section “3.2.4. General issues of bioethics,” many articles addressing this issue appear to have a common starting point: animal research plays a vital role in modern neuroscientific research. Thus, their central task is not to assess the ethical nature of animal research but to foster the public understanding of animal research from the viewpoint of better public engagement. See also Section “3.2.3. Policy and governance.” This type of neuroethical discussion, realistic in light of the actual practices of neuroscientific research, seems to have been missing in philosophical neuroethics.

### 4.3. Future research lines

This comparative study suggests some research gaps and a basis for future research. First, from the discussion in the previous subsection, some neuroethical issues have been addressed more comprehensively in the NS side of the neuroethical discourse than in its NE counterpart (or vice versa). For instance, the ethical justifiability of animal research, particularly in the context of advanced neuroscience, can be a neuroethical issue that should merit more philosophical-ethical investigation.^12^ Likewise, a bottom-up examination of the various attempts to “integrate” ethics into scientific research, focusing on actual research projects, can contribute no less productively to the neuroethical discourse among philosophical neuroethicists than among neuroscientists.

Second, some neuroethical issues have not been addressed thoroughly in either NE or NS literature but, we maintain, are worth in-depth discussion. One such underexplored issue concerns the idea of “data life-cycle management.” Although an NS article in our survey mentions it and lays out relevant ethical issues (Eke et al., 2022), more substantial discussions are required about, for instance, the ethical aspects of cross-border sharing, pseudonymization, or deletion of brain data. In particular, such a broad range of data-related issues—not limited to the oft-discussed issue of data collection with informed consent—should be analyzed from philosophical-ethical perspectives. The “life cycle” of brain data thus deserves to be one of the focal themes of neuroethics.

Another neuroethical topic that might merit more comprehensive discussion is the controversial notion of “neurorights.” Admittedly, some components of this newly proclaimed family of human rights have been mentioned both in NE (Goering et al., 2021; Bublitz, 2022) and NS (Ienca et al., 2018) articles. However, a further critical examination will be needed to see whether, and in what sense, the umbrella notion of “neurorights” is helpful for us to appreciate the fruit of neuroscience in an ethically acceptable manner.^13^

### 4.4. Limitations

Despite these intriguing observations, we should note several limitations of this study. First, a common limitation of a literature review is so-called publication bias, which leads to successful studies being published more often than unsuccessful ones (Rothstein et al., 2005).

Additionally, some might find our article selection arbitrary, as is briefly mentioned (see Section “2.1. Article selection”). Admittedly, many interesting neuroethical issues are discussed not only in specialist journals focusing on neuroethics and/or neuroscience but also in other high-reputation journals such as general journals (like *Ethics* and *Science*), bioethical journals (like *Bioethics* and *Journal of Medical Ethics*), and other neuroscientific journals (like *Current Opinion in Neurobiology* and *Neuroimage*). We believe this is an acceptable imperfection given our purpose to provide a rough comparison between the two neuroethical discourses among neuroscientists and neuroethicists. That said, there might be some bias in what is (and what is not) discussed in these specialist neuroscience/neuroethics journals, and thereby some notable articles discussing neuroethical issues may be missing in our study.

Moreover, the literature review is aimed at a qualitative comparison based on affinity diagrams developed by the authors. Thus, this study does not highlight quantitative aspects of the data—e.g., how many articles address a particular neuroethical issue and how frequently the relevant keyword appears in that article.^14^ Such a quantitative analysis can be informative to see how each neuroethical issue is discussed in the NE and NS literature; that would be a task for future research.

This study is a pilot literature review in this sense. More robust observations would be gained from future research with a quantitative method and a broader range of neuroethics-related journals.

## 5. Conclusion

Our study provides an insightful overview of the trend in which various neuroethical issues have been addressed in specialist academic journals of neuroethics and neuroscience, respectively. The two bodies of literature have significant parallelism on the major neuroethical topics covered—including diversity, open science and innovation, governance, bioethical issues, agency-influencing aspects of neurotechnology, and neuroscience-based humanities research. Besides this consistency, however, there is some difference between them regarding what neuroethical issues are highlighted and comprehensively discussed; some need more philosophical-neuroethical investigation, while others are worth more attention by neuroscientists.

Arguably, this study might be seen as a pilot literature review; more robust observations could be gained from future research with a quantitative method and a broader range of neuroethics-related journals. However, the tentative findings signal the point on which neuroethicists and neuroscientists may (dis)agree regarding various ethical issues involved in neuroscience and its potential application. This aspect would help the communication between the two academic communities (neuroscientists and neuroethicists), probably facilitating better “integration” of ethics into neuroscientific research and filling in potential lacuna of their interdisciplinary discussions. Future research with a more comprehensive nature would foster that integration.

## Author contributions

RS designed and supervised this study. HK, TT, and RS acquired the fundings. SI and YN conducted the classification of the relevant articles. SI, YN, HK, and RS extracted the data and compiled the two tables. SI and YN wrote the manuscript with comments from HK, TT, and RS. All authors contributed to the article and approved the submitted version.

## Footnotes

1 In addition to these journals, our preliminary survey also included *Nature Communications*. We omitted all articles from *Nat. Commun.* from the analysis because it is not a specialist neuroscience journal. Notably, all neuroethical issues addressed in *Nat. Commun.* articles—most of which were about the diversity in the research community—were also covered in articles from three other NS journals (*Neuron*, *Nat. Neurosci.*, or *Nat. Rev. Neurosci.*); see Section “3.2.1. Bias and diversity” below. Hence, this exclusion does not substantially affect the point of the analysis in our paper.

2 Our classification does not intend to reflect traditional taxonomies of neuroethical issues. See Levy (2009) and Roskies (2021) for alternative classifications of neuroethical issues.

3 For each neuroethical issue listed in Tables 1, 2, we hereafter pick out articles that paradigmatically address it. Notably, however, our primary concern is on the neuroethical issues instead of individual articles that address them. We do not and need not cite all 614 philosophical articles and 82 scientific articles (see Section “2.1. Article selection”). For instance, we are citing Gupta (2012) here not because it is the *only* article that addresses sexuality-based discrimination but because it is *a* paradigmatic article to address this issue.

4 Narrowly outside the scope of our article selection criterion (see Section “2.1. Article selection”), Walsh et al. (2011) in *Nat. Rev. Neurosci.* discuss ethical issues involved in neuroscientific research to identify and use biomarkers of autism spectrum disorder (ASD). They highlight the importance of respect for neurodiversity and the engagement of ASD people in relevant neuroscientific research.

5 After gathering articles as described in Section “2. Materials and methods,” Ienca et al. (2022) was published in *Neuroethics*. It addresses a wide range of issues about the ethical treatment of brain data, including data life-cycle management and data sharing.

6 Ethical issues involved in neuroeducation are not found in the NS articles in the scope of our analysis (see Section “2.1. Article selection”). An article from *Neuron*, outside the scope of our collection criterion, also deals with neuroeducation (Carew and Magsamen, 2010). However, it seldom mentions any *ethical* issue involved in neuroeducation.

7 Many of them are included in the same issue of *Neuron* (vol. 101, no. 3). Another issue of *Neuron* (vol. 92, no. 3, issued in 2016) collects worldwide case studies of neuroscientific projects in a similar manner—not only the HBP and the BRAIN Initiative. However, articles on this issue mainly focus on their scientific research in its narrow sense, rarely mentioning their attempts to address relevant ethical issues.

8 Another context in which neuroethical issues on informed consent and voluntariness are discussed is criminal justice. A typical issue concerns the moral justifiability of coerced neurointervention as a rehabilitation (Bomann-Larsen, 2013; Hübner and White, 2016; Shaw, 2019).

9 This is not to say that such criticism proves to be entirely irrelevant in neuroethics. As is discussed below, there *is* a tendency that NE articles pay much more attention to theoretical stuffs than NS articles when it comes to (say) the relationship between neuroscientific findings and human agency. See also Section “4.2. Issue-specific discussion.”

10 Compared to neuro-enhancement, the ethical issues involved in neuro-diminishment (i.e., neuroscientific intervention to reduce, rather than improve, the function of the target) are substantially discussed in neither NE nor NS articles. One reason for this could be that it is clearly problematic to reduce the capacity of others without their relevant consent. However, there is a considerable range of relevant ethical questions to be discussed, such as when and why neuro-diminishment is morally wrong. For a related notion of “mental integrity,” see Ienca (2021).

11 This is not to say that moral enhancement is out of the scope of neuroscientific research. We can point to several neuroscientific papers in an ordinary sense that report scientific findings about moral-enhancing neurotechnology, both at the basic and applied levels. Our point is that the *ethical* issues related to neuroscientific moral enhancement have not yet been covered in major NS journals, or at least not to the same degree as in major NE journals.

12 Outside the literature we employed in this study, Fenton (2014) discusses a potential criterion for ethically permissible neuroscientific experiments that involve chimpanzees.

13 In addition to seminal articles introducing the very notion of neurorights (Ienca, 2021; Yuste et al., 2021), there has been an emerging body of critical works from legal (Zúñiga-Fajuri et al., 2021; Istace, 2022) and/or ethical (Herrera-Ferrá et al., 2022; Hertz, 2022; López-Silva and Valera, 2022) perspectives.

14 In addition, our study paid no substantial attention to who wrote the relevant neuroethical articles. Perhaps it might be the case that most of the ethics-focused articles in NS journals are written by (neuro)ethicists rather than neuroscientists, which contributed to the parallelism we allegedly found in what neuroethical issues are addressed in the two bodies of literature. In that case, we admit that the relevance of our findings could be slightly limited, but only slightly. Our purpose is not to claim anything about each neuroscientist paying attention to a particular neuroethical issue but to see in general what ethical issues are shared in the neuroscientific research community. Perhaps a closer look at the “who wrote what” question might lead to an insightful observation, which is beyond the scope of this study.
